# Supplementary material for: Crop Productivity Boosters: Native Mycorrhizal Fungi from an Old-Growth Grassland Benefits Tomato (Solanum lycopersicum) and Pepper (Capsicum annuum) Varieties in Organically Farmed Soils
Source: Microorganisms. 2023 Aug 4;11(8):2012. doi: 10.3390/microorganisms11082012 (PMC10457834; doi:10.3390/microorganisms11082012)

microorganisms

Supplementary Materials

Crop Productivity Boosters: Native Mycorrhizal Fungi from an Old-Growth Grassland Benefits Tomato (*Solanum lycopersicum*) and Pepper (*Capsicum annuum*) Varieties in Organically Farmed Soils

Liz Koziol * and James D. Bever

Kansas Biological Station and Ecology and Evolutionary Biology, University of Kansas, Lawrence, KS 66047, USA
* Correspondence: lizkoziol@ku.edu

Figure S1. Cumulative log (1+g) transformed pepper biomass for Mammoth jalapeno peppers across all thirteen harvests for inoculated (black line) and non-inoculated (grey line) plants. Points are LS means from the proc mixed model and error bars are standard error.

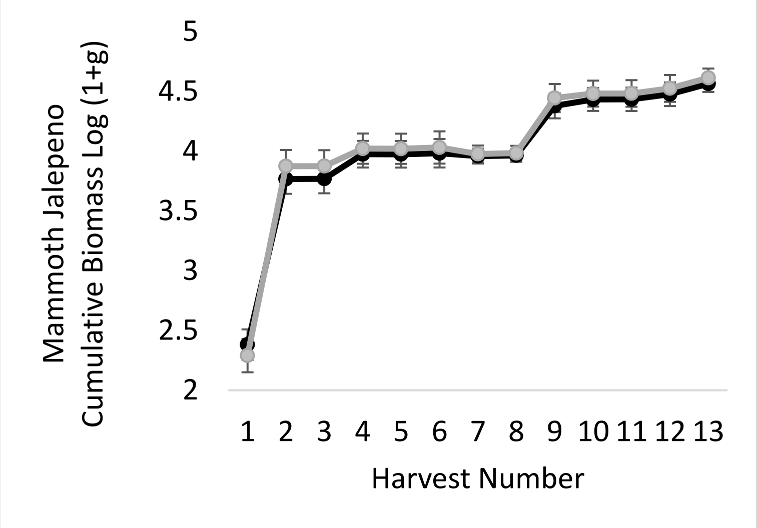

Figure S2. (**A**) total tomato biomass, (**B**) tomato number, and (**C**) average tomato size the first harvest was larger for tomato plants receiving native AM fungal inoculation (black bars) than for the non-inoculated plants (grey bars). Bars are LS means and error bars are standard error from the Proc Mixed Model outputs.

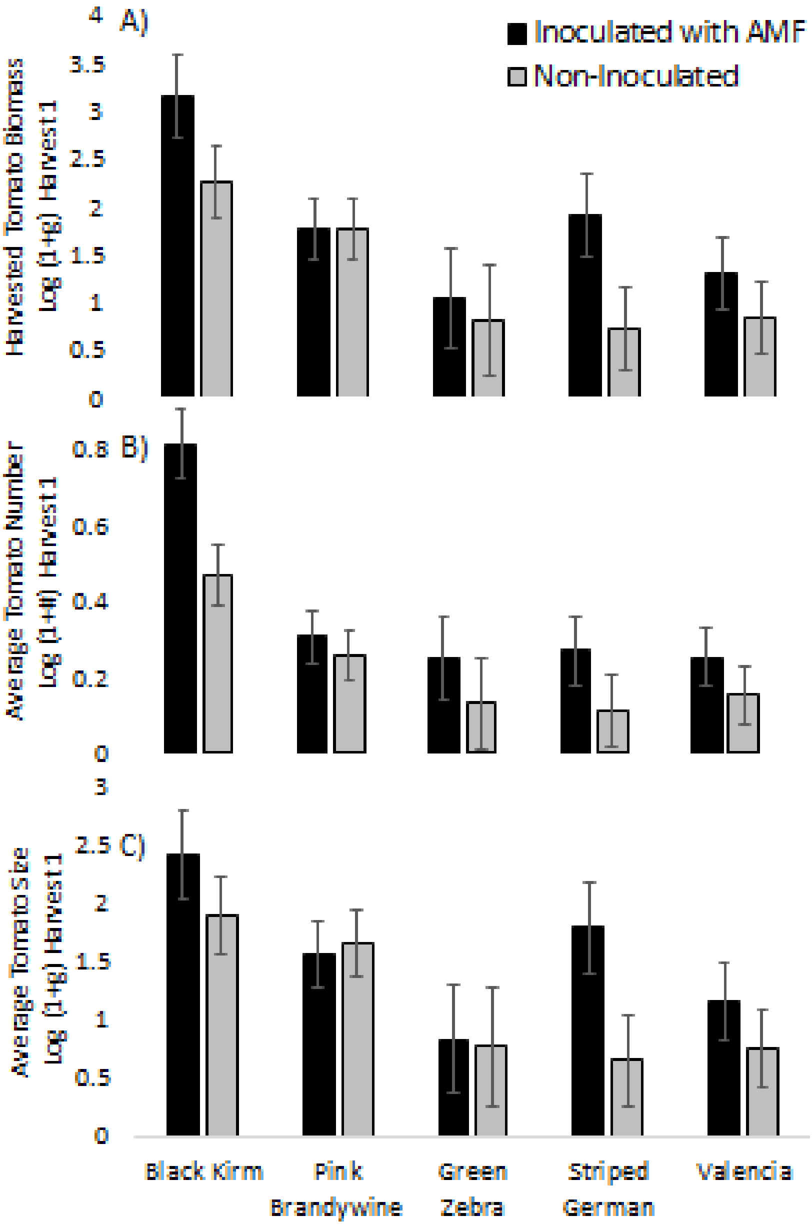

Supplement: Supplementary file 1 [file microorganisms-11-02012-s001.zip › Supplement S1 Figures.pdf]
